# Impact of integrating traditional care with the modern healthcare system in reducing tuberculosis diagnosis delays in Ethiopia: a clustered randomized controlled study

**DOI:** 10.1186/s41182-024-00641-0

**Published:** 2024-11-13

**Authors:** Desalegne Amare, Kefyalew Addis Alene, Fentie Ambaw

**Affiliations:** 1 School of Health Sciences, College of Medicine and Health Sciences, Bahir Dar University, P. O. Box: 6180/79, Bahir Dar, Ethiopia; 2 School of Population Health, Curtin University, in Bentley, Perth, WA Australia; 3 Geospatial and Tuberculosis Research Team, Telethon Kids Institute, in Bentley, Perth, WA Australia; 4 School of Public Health, College of Medicine and Health Sciences, Bahir Dar University, Bahir Dar, Ethiopia

**Keywords:** Tuberculosis, Diagnosis delay, Patient delay, Health system delay, Integration, Traditional care providers, Modern healthcare providers

## Abstract

**Background:**

Diagnosis and treatment initiation delays for tuberculosis (TB) are significant challenges in resource-limited settings. These delays can result in poor treatment outcomes, disease transmission, and increased costs. This study aimed to assess the effect of integrating traditional care with modern healthcare systems on reducing TB diagnosis delay.

**Methods:**

A cluster randomized controlled trial was conducted among TB patients, with 510 participants, 255 individuals were assigned to the intervention group and 255 to the control group. Training in the intervention group was provided for both traditional and modern healthcare providers in three rounds to enhance their knowledge, attitudes, and skills in TB screening and referral. A non-parametric independent sample test was used to compare the baseline and end-line data. The effect size was determined using Cohen’s d. To account for individual and cluster-level variations, a mixed-effect parametric survival model was employed. Furthermore, conditional (fixed only) and marginal (random effects) graphs were used to compare between the intervention and control groups.

**Results:**

A total of 510 participants were included in the baseline study, with a similar number of participants included in the endline study. In the intervention group, the delay in diagnosis was 4.185 per 1000 person-days post-intervention, compared to 4.608 per 1000 person-days pre-intervention. In the control group, the delay for diagnosis was 4.759 per 1000 person-days pre-intervention and 5.031 per 1000 person-days post-intervention. The median time to diagnosis was 135 days. The non-parametric comparison showed that the intervention significantly reduced patient delays in the intervention group compared to the control group (*p* = 0.006), with a Cohen's d effect size of 0.246. The intervention also significantly reduced diagnosis delay in the intervention group compared to the control group (*p* = 0.036), with a Cohen's d effect size of 0.187. The diagnosis of TB was accelerated by 1.076 times due to the integration of traditional care with the modern healthcare system in the intervention group compared to the control group (δ: 1.076; 95% CI 1.021, 1.134).

**Conclusions:**

The involvement of traditional care providers in TB control programs significantly reduced diagnosis delays in Ethiopia. These findings suggest the need for integrating traditional care with modern healthcare systems for the effective prevention of TB in high-burden countries.

*Clinical trial registration* ClinicalTrials.gov ID: NCT05236452.

**Supplementary Information:**

The online version contains supplementary material available at 10.1186/s41182-024-00641-0.

## Background

TB is the leading cause of death among infectious diseases worldwide, despite the availability of effective treatment for many years. In 2022, it accounted for an estimated 10.6 million new cases and 1.3 million deaths, with over 80% of cases occurring in low- and middle-income countries [1]. The Southeast Asia and Africa regions collectively account for 67% of the global TB burden, with more than 87% of new TB cases reported in 30 high-TB-burden countries, including Ethiopia [2]. Delays in TB diagnosis and treatment initiation present a significant challenge to achieving the global end-TB targets, which aim to reduce TB incidence by 90% and deaths by 95% between 2015 and 2035.

Diagnosis and treatment delays are widespread in TB control programs in low- and middle-income countries [3–5] where TB is more prevalent. In these settings, individuals with TB infections often do not receive timely diagnosis and treatment due to several factors, including the tendency of many patients first to visit traditional healthcare providers, the inaccessibility of health facilities, poverty, a lack of awareness, and poor knowledge about TB [6].

In Ethiopia, traditional medicinal practices have been used for many years. These practices, including the use of traditional medicine and holy water, focus not only on curing diseases but also on protecting and promoting the physical, mental, spiritual, social, and emotional well-being of patients [7, 8]. However, this ancient medicinal practice is not well developed and has been overshadowed by Western medicine. The lack of integration of indigenous knowledge, practices, and cultural perspectives into the healthcare system is due to weak legal enforcement, lack of government commitment and support, resource constraints, and inadequate regulatory tools. These are the main challenges that need to be addressed for traditional medicine to thrive [7, 8].

In Ethiopia, easily accessible and affordable treatment options for illnesses often include herbal and religious medicines, such as holy water. These locally available diagnosis and treatment approaches are highly accepted and trusted by the communities. Given the long history of using herbal medicine and holy water for diagnosing and treating illnesses, the scientific community should not neglect or undermine the contribution of indigenous healthcare systems. Due to the inaccessibility and unaffordability of modern healthcare systems, TB diagnosis and treatment initiation often take a long time, leading to worse clinical outcomes, continuous disease transmission, and increased TB-related costs [9–11]. For instance, previous studies have shown that more than two-thirds of TB patients in Ethiopia experience delayed diagnoses [12], with nearly 91% of TB patients taking more than 31 days for presentation, diagnosis, and treatment (referred to as total delay) [13]. Multiple factors contribute to these delays, including those related to patients, healthcare providers, and health system factors [14].

Therefore, integrating the traditional healthcare system with modern approaches is vital to reducing the time between the onset of illness and diagnosis. In Ethiopia and other low-income countries, individuals often prefer traditional care as their initial choice for TB treatment. However, the modern healthcare system lacks mechanisms to develop and retain culturally relevant TB control programs [8]. Integrating traditional care with the modern health system could help reduce diagnosis and treatment delays, leading to a more comprehensive, accessible, and patient-friendly TB care system. This integration can be achieved through effective screening, referral linkages, and targeted training, which would shorten the time from the onset of symptoms to diagnosis confirmation and treatment initiation [15–17]. This study aimed to assess the effectiveness of integrating traditional care into the modern health system to decrease TB diagnosis and treatment delays.

## Materials and methods

### Study setting

The study was conducted in the South Gondar Zone, Amhara Region, in northwest Ethiopia. The South Gondar zone comprises both town administrations and rural districts, with over 500 public health facilities at various levels, including health posts, all of which are directly or indirectly involved in the TB control program. The routine practice of the existing TB control program in this zone is passive case detection, and Direct Observation Therapy (DOT) is implemented in all public health facilities, including health posts. In addition to these modern healthcare facilities, there are several informal healthcare providers, such as traditional healers and holy water centers. We specifically selected this zone due to the widespread traditional healthcare practices.

### Study design

A cluster randomized controlled trial was conducted from April 1 to January 30, 2024, across four districts and two town administrations (totaling six districts). These districts were randomly selected from a total of 13 districts and eight town administrations. The six districts were assigned to either the intervention or control groups in a 1:1 ratio. The randomization process was carried out by experts who were not involved in the study as researchers. The number and size of the clusters within the intervention and control groups were not considered in the randomization process. All health facilities (clusters) that offer TB diagnostic and DOT programs were included in the study. Out of all the health facilities in the study area, 23 facilities were randomly selected. Of these, 13 health facilities were located at the intervention site, and 10 health facilities were assigned to the control site.

### Intervention group

The intervention group received integrated TB care that combined traditional and modern TB care services. This approach included: (1) training for both health professionals and traditional care providers; (2) TB screening at traditional healthcare sites; and (3) establishing referral linkage from traditional to modern healthcare system. The training was conducted in three rounds, aiming to increase knowledge, foster a positive attitude, and enhance skills in TB screening and patient referral activities. The intervention was designed in four phases. During the initial phase, we explored the acceptability of the intervention among various stakeholders, including traditional care practitioners, modern care practitioners, TB program planners, and clients. This was done through a qualitative study published elsewhere [18].

The second phase involved preparing comprehensive training manuals that were reviewed and standardized by invited experts, including physicians, public health specialists, nurses, and language experts. A workshop was conducted to refine the training manuals, with feedback provided on the content, depth, readability, and comprehensibility. The manuals covered various aspects of TB, including causes, symptoms, signs, transmission, screening and referral procedures, diagnostic approaches, case detection techniques, treatment outcomes, the benefits of early detection, the challenges of late diagnosis, and TB control and prevention strategies. The training also included models for integrating traditional and modern healthcare systems, which were approved by senior experts.

In the third phase, training was provided to traditional practitioners such as traditional healers and religious leaders in a five-day training session, while healthcare providers at DOT clinics and TB focal persons received two days of training. Additional one-day sessions were held three and six months after the initial training. The training was led by researchers and certified TB experts. Traditional healers and religious leaders who showed proficiency in knowledge, attitude, and skills through a post-training test and practical assessment were included in the intervention to screen and refer presumptive TB cases. Details of the operational procedures for the intervention packages are provided in a published protocol [19] and are available in the supplementary materials (S1-Table S1). The intervention was fully implemented when patients were screened and referred to nearby health facilities by traditional healers and religious leaders using standardized screening tools. Suspected TB cases were referred to health facilities in the intervention districts, where trained TB focal persons re-screened and diagnosed patients according to national TB treatment guidelines. Regular supervision was conducted to ensure the effective implementation of the intervention.

In the final phase, the end-line outcome was assessed by comparing the differences between the end-line and baseline results in both the intervention and control groups. The details of screening and referral formats are provided in the supplementary materials (S2: screening form and S3: referral format).

### Control group

In the control group, routine TB care continued without any additional intervention from the research team. In Ethiopia, routine TB care involves identifying individuals with TB when they independently seek healthcare services due to symptoms or other health concerns. The control group served as a reference comparator for measuring the effectiveness of integrated interventions. Baseline information was collected simultaneously in both the control and intervention groups.

### The outcome of interest and its definition

The outcome interest of this study was the delay in diagnosis and treatment commencement for TB. Delays in TB diagnosis include patient delay, health system delay, and treatment delays. Diagnosis delay is defined as the time interval between the onset of symptoms and the confirmation of TB in the patient. Patient delay refers to the period from the onset of the first symptom to the first medical consultation [20]. Health system delay is defined as the time from the first consultation to the date of TB diagnosis [20]. Treatment delay refers to the time from diagnosis to the start of TB medication [21]. Detailed definitions of the outcome of interest and other variables are provided in the supplementary materials (S4-Table S2). The median diagnosis time was used as the cutoff point to determine whether the diagnosis was delayed [3, 22]. In this study, any time exceeding the median value was classified as a “delayed diagnosis”, while time below the median was considered “not delayed”.

### Participant recruitment and randomization

Two districts and one town administration were assigned to the intervention group, while the other two districts and one town administration were assigned to the control group. A total of 23 public health facilities within the intervention and control districts were included in the study. Additionally, 29 traditional care centers located at the intervention site were selected for implementing the intervention. Using a random sampling approach, Dera woreda, Libokemekem woreda, and Worta town were chosen as intervention districts, while Farta, Gunabeyemeder, and Debre-Tabor town were selected as control districts. The districts and town administrations were randomly allocated to the intervention and control groups. In the study area, the level of training for providers, DOT programs, laboratory supplies, diagnostic techniques, and guidelines were consistent across all health facilities. Study participants were selected randomly. To minimize information contamination, a distance of more than 10 km was maintained between the intervention and control groups.

### Data safety and adverse effects monitoring

The trial involved the integration of traditional care with modern care without the use of invasive procedures or the administration of any drugs. Participant adherence in both intervention and control groups was monitored through self-reports and direct observation by trained field supervisors, with regular communication and feedback maintained between supervisors and researchers. Ethical approval was obtained from the institutional review board (IRB) of Bahir Dar University, College of Medicine and Health Sciences, with ethical review Ref No. 353/2021. Written consent was obtained from all adult participants, and written assent was obtained for pediatric participants.

### Sample size determination

The sample size was determined using a two-sample comparison of means, based on data from a previous study on diagnosis and treatment delays in southwestern Ethiopia, which reported a median delay of 55 days (interquartile range (IQR): 32–100) (median: m1 = 55) [23]. We assumed a 14-day reduction in diagnosis delay in the intervention group compared to the control group (m2 = 41). Considering a type 1 error probability of 0.05, a 95% confidence interval, 80% power, and accounting for a 10% non-response rate, the initial sample size was calculated to be 537 participants. Since the study design is a cluster randomized controlled trial, a design effect was applied. The design effect was determined to be 0.95, based on a recommended intraclass correlation coefficient (ICC) of less than 0.052 for studies with more than 10 clusters. After multiplying the initial sample size by the design effect, a total of 510 samples were included in the baseline study, with 250 in each intervention and control group. Similar sample sizes were included in the end-line study.

### Data collection

Data were collected using structured interview questionnaires. The data included information on socio-demographic and socio-economic status, clinical characteristics, onset of symptoms, health-seeking behavior, and first consultations obtained by interviewing TB patients. Other information, such as the date of diagnosis and treatment commencement, was collected from medical records. The data were collected by nurses and public health officers who had training and relevant experience in data collection. The data collection process is also supervised by experts who hold a master’s degree in public health.

### Statistical analysis

Data were entered into EpiData version 4.6 and exported to Stata software version 17.0 for analysis. The baseline data were analyzed using descriptive statistics, and the household wealth index was constructed using principal component analysis based on variables such as livestock and land ownership. Mean and standard deviation were used to compare continuous variables like age between the intervention and control groups. The characteristics of the baseline and end-line data were compared between the intervention and control groups using the Chi-square test for categorical variables and the independent t-test for continuous variables. A non-parametric Mann–Whitney U test was used instead of parametric testing to compare the baseline and end-line data for each group, and the effectiveness of the intervention was assessed using Cohen’s d, which was calculated based on Lenhard W and Lenhard A (2022) recommendations [24].

The unit of measurement for the outcome variable was “days”, which indicated the length of delays from the onset of symptoms to diagnosis; it was suitable for survival regression analysis. This was presented as person-days to incorporate the time component and the data included individual and population (facility) level variables. A two-level mixed-effects parametric survival regression model was utilized to examine the influence of facility factors on the outcome variable, with diagnosis delay at level 1 nested within facilities at level 2. The analysis consists of four steps using four models: Model 1 (the null model) was fitted without explanatory variables to evaluate the random variability in the intercept and show the total variance in diagnosis delay among TB patients in different facilities. Model 2 examined the effect of individual-level characteristics. Model 3 examined the effect of facility-level variables, and Model 4 (full model) analyzed the influence of both individual and facility-level characteristics. In addition, the impact of the intervention on the time to diagnosis delay was assessed graphically using conditional (fixed only) and marginal (random) methods.

The two-level mixed-effect parametric survival model with a binary response for TB patients i, living in community j, is represented as log [hij (t)] = log[h0(t)] + XT ijβ + ZTi bi, where h0(t) represents the baseline hazard function of a parametric distribution of the log-normal. We define design matrices Xij and Zi for the fixed (β) and random (bi) effects, respectively.

The Cox proportional hazards assumption was assessed using both graphic and statistical methods, and it was found to be met in both cases. The Schoenfeld residual test yielded a p-value of 0.61. The outcome variable was measured in days, representing the length of delays from symptom onset to diagnosis, making it suitable for survival regression analysis. This was presented as person-days to incorporate the time component, and the data included variables at both the individual and population (facility) levels. Additionally, we compared survival distribution and parameterization for model fitness. The log-normal accelerated failure time (AFT) model, which had the lowest Akaike information criteria (AIC) and Bayesian information criteria (BIC) values, was selected for analysis. To aid in understanding and interpretation, the beta coefficient was exponentiated and interpreted as the accelerated failure time denoted by (δ). The significance level of the accelerated failure time was determined at a 95% confidence interval with p < 0.05.

## Results

### Cox–Snell residuals plot

**Fig. 1:**
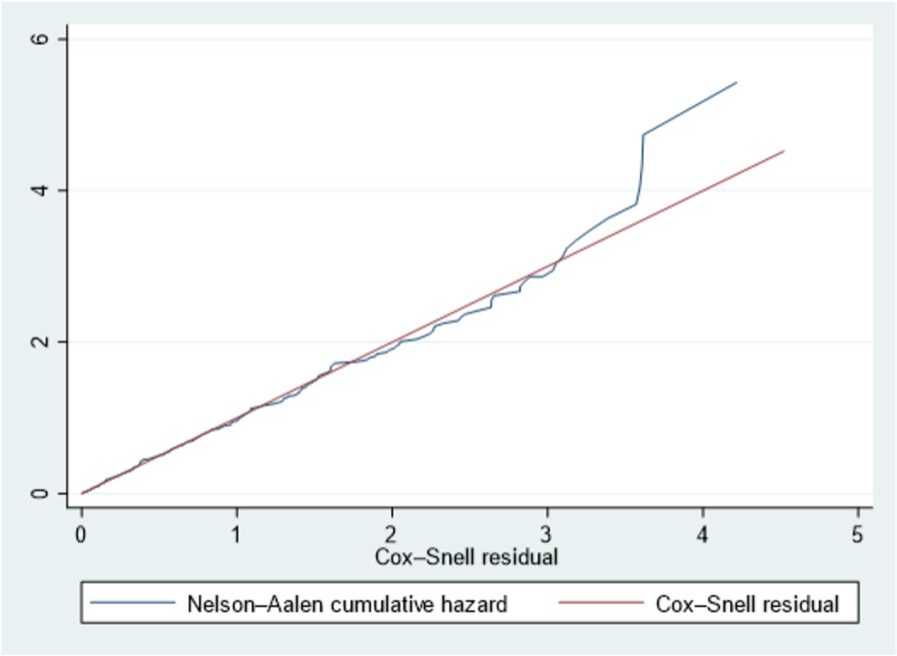
Cumulative hazard plot of Cox–Snell residual for the AFT model

The Cox–Snell residuals plot showed that the model could effectively predict the time to diagnosis delay. It closely followed the 45-degree straight line, with a slight deviation noted on the left tail (Fig. 1).

### Socio-demographic characteristics

A total of 510 patients diagnosed with TB were included in the baseline study: 255 in the control group and 255 in the intervention group. A similar number of patients were included in the end-line study, with 255 participants in each group.

The mean (SD) age of participants was 36.2 (± 16.4) years in the intervention group and 41.4 (± 15.6) years in the control group in the baseline study. Similarly, in the end-line study, the mean age was 40.6 (± 14.5) in the control group and 38.4 (± 17.7) in the intervention group.

In the baseline data, there were 93 cases of smear-positive PTB, 71 cases of smear-negative PTB, and 91 cases of EPTB in the control group. In the intervention group, there were also 49 cases of smear-positive PTB, 53 cases of smear-negative PTB, and 153 cases of EPTB.

In the end-line data, the control group had 89 cases of smear-positive PTB, 78 cases of smear-negative PTB, and 88 cases of EPTB. The intervention group had 52 cases of smear-positive PTB, 59 cases of smear-negative PTB, and 144 cases of EPTB.

In the baseline data, the mean distance from home to the health facility was 4.7 (± 3.6) kilometers in the intervention group and 4.2 (± 2.8) kilometers in the control group, while in the end-line data, the mean distance was 4.7 (± 3.5) kilometers in the control group and 4.3 (± 2.9) kilometers in the intervention group.

There was no statistical difference between the intervention and control groups in alcohol consumption, cigarette smoking, and drug use in both the baseline and end-line data (Table 1).

**Table 1:** Baseline and end-line socio-demographic, behavioral, and clinical characteristics of study participants in northwest Ethiopia

Variables	Baseline			End-line		
	Control group (*n* = 255)	Intervention group (*n* = 255)	*P*-value	Control group (*n* = 255)	Intervention group (*n* = 255)	*P*-value
Socio-demographic characteristics						
Sex			0.091			
Male	149	130		150	134	0.154
Female	106	125		105	121	
Residence			0.595			
Urban	127	121		124	133	0.425
Rural	128	134		122	131	
Age in year	41.4(± 15.6)	36.2(± 16.4)	0.065	40.6(± 14.5)	38.4(± 17.7)	0.142
Distance from home to health facility in kilo meter	4.2(± 2.8)	4.7(± 3.6)	0.120	4.7(± 3.5)	4.3(± 2.9)	0.138
Behavioral characteristics						
Alcohol drinking						
Yes	7	11	0.337	5	11	0.127
No	248	244		250	244	
Substance use (smoking)						0.704
Yes	6	2	0.154	4	3	
No	249	253		251	252	
Drug user						
Yes	4	2	0.411	3	4	0.704
No	251	253		252	251	
Clinical characteristics						
Types of TB						
PTB +	93	49	0.01	89	52	0.01
PTB−	71	53		78	59	
EPTB	91	153		88	144	
TB category						
New	248	246	0.611	249	248	0.779
Relapse	7	9		6	7	
HIV status						
Positive	26	20	0.354	28	21	0.293
Negative	229	235		227	234	
Lung disease other than TB						
Yes	7	6	0.779	6	8	0.588
No	248	249		249	247	
Co-morbidity						
Yes	17	16	0.857	18	17	0.61
No	238	239		237	238	

PTB +,  pulmonary positive tuberculosis, PTB−, pulmonary negative tuberculosis, EPTB,  extrapulmonary tuberculosis

### Diagnosis and treatment delays

A Mann–Whitney U test was conducted to compare the difference between the intervention and control groups following the intervention. The results of the Mann–Whitney *U* test indicated that patient delay, diagnosis delay, and total care delays were significantly decreased in the intervention group compared to the control group. Specifically, patient delay was significantly reduced in the intervention group (*U* = 27,926.50, *z* = − 2.757, *p* = 0.006, Cohen’s d = 0.246) compared to the control group (*U* = 30,525.50, *z* = − 1.195, *p* = 0.232, Cohen’s d = 0.246). However, there was no significant reduction in health system delay after integrating traditional care with modern care in either the intervention or control groups. The diagnosis delay was significantly reduced in the intervention group (*U* = 29,018.00, *z* = − 2.100, *p* = 0.036, Cohen’s d = 0.187) compared to the control group (*U* = 31,881.50, *z* = − 0.37, *p* = 0.705, Cohen’s *d* = 0.034). Additionally, the intervention was significantly associated with a reduction in total delay in the intervention group (*U* = 29,141.50, *z* = − 2.026, *p* = 0.043, Cohen’s d = 0.180) compared to the control group (*U* = 32,120.00, *z* = − 0.24, *p* = 0.814, Cohen’s d = 0.021) (Table 2).

**Table 2:** Mann–Whitney *U* test to compare the diagnosis and treatment delay before and after the intervention between the intervention and control groups in northwest Ethiopia

Variables	Control group (*n* = 255)				Effect size Cohen’s d	Intervention group (*n* = 255)				Effect size Cohen’s d
	Rank mean		Mann–Whitney *U*	Z (*p*-value)		Rank mean		Mann–Whitney *U*	Z (*p*-value)	
	Before	After				Before	After			
Patient delay	263.29	247.71	30,525.50	− 1.19 (0.232)	0.106	273.48	237.52	27,926.50	− 2.757 (0.006)	0.246
Health system delay	248.12	262.88	30,629.50	− 1.13 (0.258)	0.100	261.94	249.06	30,869.50	− 0.987 (0.323)	0.088
Diagnosis delay	257.97	253.03	31,881.50	− 0.37 (0.705)	0.034	269.20	241.80	29,018.00	− 2.100 (0.036)	0.187
Treatment delay	259.08	251.92	31,600.00	− 0.61 (0.543)	0.049	256.20	254.80	32,335.00	− 0.115 (0.908)	0.009
Total delay	257.04	253.96	32,120.00	− 0.24 (0.814)	0.021	268.72	242.28	29,141.50	− 2.026 (0.043)	0.180

### Time to TB diagnosis among the study population

The median time to diagnosis was 135 days (95% CI: 102, 223). In the intervention group, the delay in diagnosis was 4.185 per 1000 person-days (95% CI: 3.656, 5.031) post-intervention, compared to 4.608 per 1000 person-days (95% CI: 3.970, 5.349) pre-intervention. During the post-intervention period, the total time at risk and under observation was 27,358 days, with a total follow-up time of 107,708 days. In comparison, during the pre-intervention period, there were 37,538 at risk under observation, with 147,787 days of follow-up time.

In the control group, the delay for diagnosis was 4.759 per 1000 person-days (95% CI: 4.035, 5.614) pre-intervention and 5.031 per 1000 person-days (4.233, 5.979) post-intervention.

### Effect of the intervention on diagnosis delay of TB

**Fig. 2:**
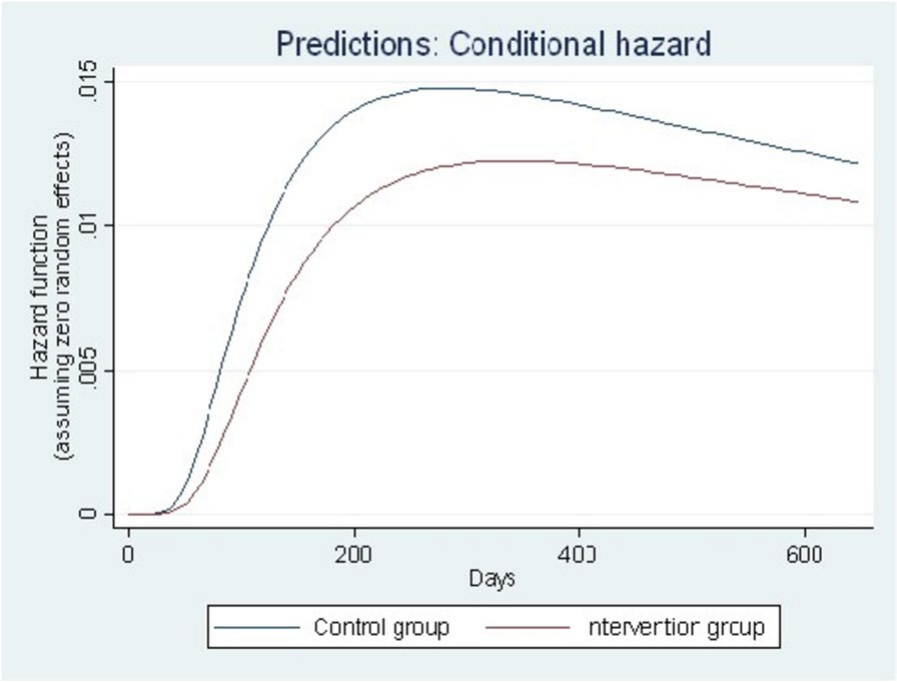
Conditional hazard function with only fixed effect

**Fig. 3:**
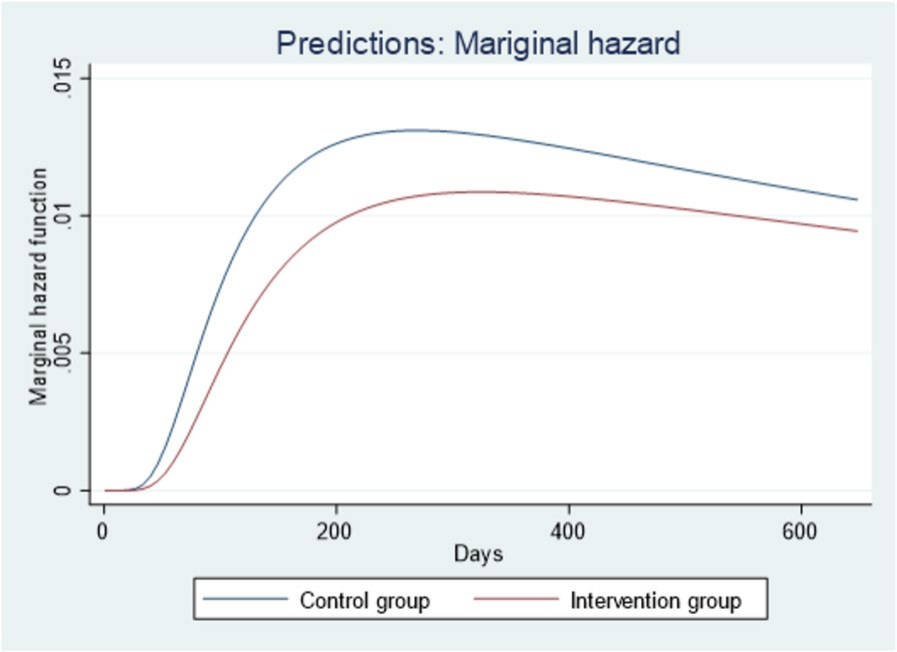
Marginal hazard function with random effect distribution

Figure 2 shows that the time to TB diagnosis in the control group was significantly higher than the time to TB diagnosis in the intervention group, as shown in the fixed-only conditional prediction graph with zero random effects. Similarly, Fig. 3 reveals that the time to diagnosis was notably longer in the control group compared to the intervention group, as shown in the population-level prediction (marginal hazard function) graph with random effects.

The variance of the individual-level residual errors (the variability of the average diagnosis delay within individuals) was 5%, which was statistically significant (p < 0.05). Factors including age, sex, residency, educational level, occupation, household wealth index, knowledge, diagnostic centers, and distance from home had no acceleration or deceleration on the time to diagnosis of TB. After controlling confounders, diagnosis of TB was accelerated by 1.076 times in the intervention group compared to the non-intervention group (δ: 1.076; 95% CI 1.021, 1.134) (Table 3).

**Table 3:** Mixed effect parametric survival regression model on predictors of time to TB diagnosis in northwest Ethiopia

Variable	Model 1		Model 2		Model 3		Model 4	
	Estimate (SE)	95% CI	Estimate (SE)	95% CI	Estimate (SE)	95% CI	Estimate (SE)	95% CI
Intercept	162.065 (1.058)	144.893,181.272	60.714 (1.075)	52.610,70.067	65.117(1.051)	58.975, 71.899	60.281(2.285)	51.266,70.880
Pre-intervention			1.001(1.001)	0.999, 1.001	1.000(1.000)	0.999, 1.001	1.000(1.0001)	0.999,1.001
Post-intervention*			1.005(1.001)	1.004,1.006	1.005(1.000)	1.004, 1.005	1.0046(1.001)	1.004, 1.005
Intervention effect*			1.084(1.025)	1.017, 1.125	1.080(1.026)	1.027, 1.136	1.076 (1.027)	1.021, 1.134
Age in year			1.001(1.000)	0.999, 1.002			1.001(1.001)	0.999, 1.002
Sex								
Male			Ref.				Ref.	
Female			1.018(1.021)	0.977, 1.061			1.033(1.019)	0.999, 1.073
Educational level								
Unable to read and write			Ref.				Ref.	
Primary school			0.960(1.028)	0.908, 1.015			0.967(1.025)	0.919, 1.018
Secondary school			0.966(1.043)	0.888, 1.051			0.956(1.041)	0.884, 1.034
Above high school			0.965(1.040)	0.892, 1.043			0.969(1.038)	0.900, 1.044
Occupation								
Employed			Ref.				Ref.	
Housewife			1.033(1.046)	0.944, 1.129			1.138(0.932)	0.932, 1.101
Students			1.001(1.043)	0.921, 1.087			0.991(1.041)	0.914, 1.073
Farmer			0.963(1.037)	0.897, 1.034			0.946(1.036)	0.882, 1.015
Daily worker			1.042(1.061)	0.927, 1.170			1.037(1.056)	0.931, 1.155
Merchant			0.986(1.047)	0.986, 1.080			0.984(1.044)	0.904, 1.071
Distance in km			0.997(1.002)	0.991, 1.003			0.998(1.003)	0.993, 1.004
Knowledge								
Poor knowledge			Ref.				Ref.	
Good knowledge			1.068(1.041)	0.986, 1.156			1.064(1.037)	0.991, 1.142
Wealth index								
Poorest			Ref.				Ref.	
Poor			0.995(1.031)	0.934, 1.716			0.995(1.030)	0.939,1.055
Middle			1.011(1.034)	0.946, 1.081			1.003(1.031)	0.943, 1.066
Rich			1.029(1.032)	0.966, 1.095			1.0.25(1.031)	0.966, 1.089
Richest			0.975(1.034)	0.913, 1.042			0.974(1.033)	0.923, 1.050
Residence								
Urban					Ref.		Ref.	
Rural					0.989(1.020)	0.951, 1.029	0.973(1.021)	0.934, 1.015
First visiting facility								
Traditional care center					Ref.		Ref.	
Public health facility					0.994(1.026)	0.945, 1.046	0.981(1.025)	0.934, 1.029
Private health facility					0.925(1.046)	0.846, 1.123	0.950(1.042)	0.875, 1.031
Diagnostic center								
Health center					Ref.		Ref.	
Public hospital					1.019(1.038)	0.946, 1.018	1.034(1.035)	0.965, 1.108
Private hospital/clinic					1.018(1.044)	0.934, 1.109	1.033(1.042)	0.952,1.121

Random effect								
Variation (σ)	0.05(0.02)	0.01(0.01)	0.001(0.001	0.02(0.01)				
AIC	2712.927	2168.408	2168.118	2171.542				
BIC	2725.63	2261.522	2214.697	2285.818				

*Variables that significantly accelerated the diagnosis of TB after intervention

SE: standard error, CI: confidence interval, AIC: Akaike information criteria, BIC: Bayesian information criteria

## Discussion

The purpose of this study was to investigate the effectiveness of integrating traditional care with modern healthcare systems to reduce diagnosis and treatment delays in TB burden and resource-limited settings. The duration from the onset of TB symptoms to diagnosis and treatment commencement is often prolonged in resource-limited countries [25]. Previous studies have identified various factors associated with diagnosis and treatment delays [3, 26, 27]. However, there are still several factors yet to be investigated, particularly the role of traditional healthcare practices and their integration with modern healthcare systems in reducing these delays. In our experience, many people in Ethiopia continue to seek diagnosis and treatment from traditional healers and holy water, a practice that has persisted for many years [7, 8]. This implies that relying solely on biomedical approaches may not effectively reduce TB diagnosis and treatment delays. Integrating traditional medicine with modern healthcare is essential for improving these timelines and patient outcomes. Studies have shown that traditional healers are willing to cooperate with national TB control programs, and with proper training and support, they can play a key role in referring undiagnosed TB cases to modern healthcare facilities [28–31].

Our study is the first to investigate the impact of integrating traditional care providers with modern healthcare systems to reduce diagnosis and treatment delays. The integration significantly reduced patient delays, with a standardized mean difference (Cohen’s d) of 0.246 in the intervention group compared to the control group. Similarly, the time to diagnosis delay decreased by 0.187 in the intervention group compared to the control group.

Although the method used in this study was rigorous in controlling confounders and errors, such as random error, selection, and information bias, leading to increased reliability and validity of the study, it is important to note that our study was conducted in a specific geographical region with unique cultural practices. This may limit the generalizability of our findings. In addition, some participants experienced recall bias regarding the symptoms of their sickness, as they were unable to remember the exact date when they first occurred. Addressing prolonged diagnosis delays remains challenging in resource-limited settings and countries with weak healthcare systems, such as Ethiopia. Despite these limitations, we believe that engaging the community and involving traditional healers and religious leaders in TB control activities can be a valuable approach to reducing long-term delays in TB diagnosis and treatment initiations.

A previous study in a similar setting showed that integrating traditional care with the modern healthcare system was well-accepted by various stakeholders, which could enhance the effectiveness and sustainability of implementation [18]. Therefore, integrating traditional care with the national TB program is important for reducing patient and diagnosis delays and limiting disease transmission within the community. In our study, this strategy was effective in reducing both patient and diagnosis delays.

Based on our findings, we recommend that TB programmers and policymakers implement and expand this intervention in countries with similar contexts. We believe that including traditional healers and religious leaders in TB care is essential for reducing long-term delays in TB diagnosis in resource-limited and high-TB-burden settings, as many patients seek care outside modern healthcare systems.

## Conclusions

The study found that integrating traditional care with modern healthcare systems significantly reduces delays in TB diagnosis and treatment. This approach involves traditional healers and religious leaders in TB screening and linkage activities, which is important for improving TB outcomes in high-burden settings such as Ethiopia.

## Supplementary Information

Additional file 1: Table S1. Operational Definitions, S2. Screening format; S3. referral format; S4-Table S2. description of outcome of interests.

## Data Availability

The data in this study are available upon request from the corresponding author.

## Abbreviations

AFT: Accelerated failure time
AIC: Akaike information criteria
BIC: Bayesian information criteria
CI: Confidence interval
DOT: Direct observation therapy
SE: Standard error
EPTB: Extrapulmonary tuberculosis
PTB: Pulmonary tuberculosis
TB: Tuberculosis
