# Supplementary material for: Impact of integrating traditional care with the modern healthcare system in reducing tuberculosis diagnosis delays in Ethiopia: a clustered randomized controlled study
Source: Trop Med Health. 2024 Nov 13;52:83. doi: 10.1186/s41182-024-00641-0 (PMC11558963; doi:10.1186/s41182-024-00641-0)
Supplement: Supplementary file 1 — Additional file 1: Table S1. Operational Definitions, S2. Screening format; S3. referral format; S4-Table S2. description of outcome of interests. [file 41182_2024_641_MOESM1_ESM.docx]

**Supplementary files**

S1-Table 1: Intervention packages, methods frequency, duration, dosage, and components of the intervention

| Activities | Methods | Frequency, duration, and doses | Contents of the training manuals |
| --- | --- | --- | --- |
| Training for both categories of practitioners (traditional and modern care providers) | Group-based training was provided by researchers | - The training was provided three times - 1^st^ round for five days - 2^nd^ round for 1 day at 3^rd^ months - 3^rd^ round for 1 day at 6^th^ months | - Training manual for traditional care providers includes the following contents: - To increase the knowledge of traditional care providers the following contents were included: - Definition of TB, - Causes, - Signs and symptoms, - Mode of transmission, - Prevention and control, - Advantage of early TB diagnosis and treatment, - Health and economic impact of TB on the families and the community, and - Integration of traditional and modern healthcare systems. - To create a favorable attitude toward traditional care providers the following contents were included: - Healthcare providers’ cooperativeness towards integration, - Healthcare providers' attitudes towards traditional healers and religious leaders, - Positive perspective of healthcare providers towards herbal medicine and holy water, - Healthcare providers believe and respect religious leaders and traditional healers. - To acquire skills in screening and referring patients, we included contents of medical history taking, inspection of the overall general appearance of the patient, and ability to identify lymphadenopathy through palpation. - **Training manual for modern care providers**   To increase the knowledge of modern healthcare providers the following contents were included:   - Global and national burden of TB, - Evidence for low case detection rate - Challenges to increase case detection rate, - The role of traditional care in increasing case notification and integration of modern healthcare with the traditional healthcare system to increase the accessibility of alternative healing facilities and patient-centred care were included. - The training was implemented through dictating, group discussion, case scenario, and simulative exercise, video and role play to increase the skill of practitioners. |
| TB screening | - Subjective and objective data were gathered - Role-play sessions were performed | Screen patients every case for every day through one year | - To crate favorable attitude of healthcare providers the following contents were included: - Acceptance of providing drugs and holy water simultaneously by religious leaders - Cooperativeness of traditional care providers with healthcare providers - Healthcare providers believe and respect religious leaders and traditional healers |
| Screening patients who need spiritual support by healthcare providers | Group-based training and demonstration and role-play of how healthcare providers screen patients who need spiritual support | Screen patients every case for every day through one year | Use any signs and symptoms of stress, such as sadness, frustration, or emotional instability, as an indicator. |
| Referral linkage | Group-based training and individual-based practical/demonstration and role-play sessions were conducted on how to refer suspected patients | Refer patients every case for every day through one year | - Patient identification - Coughing for two weeks or   at least two sign and  symptoms of TB infection |

S2-TB screening form

Name of the patient: __________________________

Date of visiting ________________ Address _________________town___________Telephone Number___________________

Screening date: _____________________________

| Signs and symptoms | Yes | No |
| --- | --- | --- |
| Any cough for two weeks more than two weeks |  |  |
| Persistent fever |  |  |
| Night sweating |  |  |
| Unintentional weight loss (if children, failure to weight gain, failure to thrive and malnutrition) |  |  |
| History of contact |  |  |
| History of previous TB treatment |  |  |
| Chest pain |  |  |
| Persistent shortness of breath (Dyspnea? |  |  |
| Weakness or fatigue |  |  |
| No appetite |  |  |
| Chills |  |  |
| Hemoptysis |  |  |
| Lymph node enlargement (lymphadenopathy) |  |  |
| Have you had contact with anyone having a cough or tuberculosis disease in the past year? |  |  |
| Do you have a medical condition, or are you taking medications? |  |  |
| Have you had contact with a family member or partner who has been diagnosed with TB/having coughed? |  |  |

If any of the above answers are "yes", instruct the client to wear the mask and refer for further evaluation to the nearby health institutions.

Commentes:_________________________________________________________________________________________________________________________________________________________________________________________________________________________________________________________________

**S3-referral form**

Referral form used to screen presumptive TB cases by traditional care practitioners

Name of presumptive TB cases: ____________________________________________

Date of visiting ________________ Address _________________town___________

Telephone number if (available): ___________________

Screening date: ________________________________

Presenting signs and symptoms:

1._______________________2. ______________________3.______________________

4._______________________5.______________________6._______________________

Duration of symptoms for: _______________________________days/weeks/months/years

Date of refer _______________________________

Referral code: _______________________________

Refer from (Name of visiting traditional center)______________to _______________ health center/hospital

Comments:____________________________________________________________________

Name of the evaluator: _____________________________ Signature: _________________

S4-Table2

**Table S2:** **Definitions of the outcome of interest and other variables**

| Terms | Description of terms |
| --- | --- |
| Patient delay | The term “patient delay” is the time interval between the onset of symptoms and presentation to health care providers. |
| Health system delay | The term” health system delay” is the time interval between the date of health-seeking behavior at a health care provider and the initiation of anti-TB treatment. |
| The diagnosis delay | “Diagnosis delay” is the time interval between the onset of symptoms and the confirmation of TB diagnosis, which includes patient delay and health system delay. |
| Treatment delay | The time interval between the TB diagnosis and the initiation of anti-TB drugs |
| The total delay | The term “total delay” is the time interval from the onset of illness until the initiation of anti-TB drugs. Total delay is the sum of diagnostic delay and the treatment delay or the total delay is the sum of patient and healthcare system delays. |
| Integrating traditional TB care with modern care | The term "integration of traditional tuberculosis care with modern care services" is not well defined. However, for the sake of this study, integrating traditional care with modern TB care means working collaboratively using referral linkage and patient screening between traditional and modern care providers. |
| Traditional healer | A healer is a person who seeks to cure diseases or heal injuries by means other than conventional medical treatment. |
| Traditional medicine | Traditional medicine is the sum of the total of the knowledge, skills, and practices based on the theories, beliefs, and experiences indigenous to different cultures, whether explicable or not, used in the maintenance of health as well as in the prevention, diagnosis, improvement, or treatment of physical and mental illness. |
| Holy water | Holy water is water that has been blessed by a member of the clergy and used in baptism to bless individuals, churches, homes, and articles of devotion. |
